# S84. NEUROPSYCHOLOGICAL FUNCTIONING AS A PREDICTOR OF PSYCHOLOGICAL RESILIENCE: PRELIMINARY RESULTS FROM THE PRONIA STUDY

**DOI:** 10.1093/schbul/sby018.871

**Published:** 2018-04-01

**Authors:** Alexandra Stainton, Katharine Chisholm, Rachel Upthegrove, Stephen Wood, PRONIA Study Group

**Affiliations:** 1 University of Birmingham; 2 Orygen, the National Centre of Excellence in Youth Mental Health

## Abstract

**Background:**

Resilience provides a new understanding of the highly variable trajectories of mental illness, and has consistently been linked with improved mental health outcomes. Resilience is largely defined as the presence of additional factors which overcome a specific risk for mental illness, leading to ultimately more positive outcomes than expected given said risk. Previous research in the area has focused on identifying psychological factors which may be associated with resilience. Moving forwards, it is essential that researchers investigate how resilience may function in different domains. The aim for the present research was to conduct a preliminary investigation into the possible role of neuropsychological performance in resilience using data from the PRONIA study.

**Methods:**

Participants were individuals aged 15–40 who were recruited into the PRONIA study. Total scores for the Resilience Scale for Adults (RSA), assessing self-report psychological resilience, were available for 587 participants. The sample included individuals with first-episode psychosis (N=113), first-episode depression (N=118), individuals at ultra-high risk for psychosis (N=109), and healthy controls (N=247). Participants also completed a comprehensive neuropsychological test battery which assessed performance in the following domains: IQ, executive functioning (EF), processing speed (PS), sustained attention, working memory, visual memory, social cognition, motivational salience, and verbal learning and memory.

**Results:**

A stepwise multiple linear regression was used to identify which of the neuropsychological domains would best predict RSA total score. The final model significantly predicted RSA total score, explaining 4% of the variance in these scores, F(2, 512) = 12.37, p < 0.001. The model indicated that higher RSA total was associated with PS (β=3.35, p=.032) and EF (β=4.15, p=0.046). EF provided the highest relative contribution in the model, with every 1 point increase resulting in 4.15 standard deviation increase in RSA total.

**Discussion:**

The present results suggest that neuropsychological performance has a small, but significant relationship with psychological resilience. The two neuropsychological domains which best predicted this outcome were PS and EF. Resilience has been argued to be a highly dynamic process, by which individuals must utilise assets and resources to their benefit. Furthermore, the effectiveness of such factors will vary across time and circumstance, adding to the flexibility required to navigate this process. These results support this conceptualisation of resilience, as EF is thought to involve the organisation and execution of complex thoughts and behaviour. Processing speed has also been found to affect other cognitive functions such as reasoning. These neuropsychological processes may aid an individual’s ability to utilise protective factors to their benefit during a period of adversity or risk. These results are preliminary, and future research should look to replicate and extend this research to form a multi-modal model of resilience. A deeper understanding of the mechanisms underlying this process can then inform future intervention strategies.

